# Regulation of autophagic cell death by glycogen synthase kinase-3β in adult hippocampal neural stem cells following insulin withdrawal

**DOI:** 10.1186/s13041-015-0119-9

**Published:** 2015-05-19

**Authors:** Shinwon Ha, Hye Young Ryu, Kyung Min Chung, Seung-Hoon Baek, Eun-Kyoung Kim, Seong-Woon Yu

**Affiliations:** Department of Brain and Cognitive Sciences, Daegu Gyeongbuk Institute of Science and Technology (DGIST), Daegu, 711-873 Republic of Korea; College of Pharmacy, Ajou University, Suwon, 443-749 Republic of Korea; Neurometabolomics Research Center, Daegu Gyeongbuk Institute of Science and Technology (DGIST), Daegu, 711-873 Republic of Korea

**Keywords:** Hippocampal neural stem cells, Programmed cell death, Autophagic cell death, Glycogen synthase kinase-3β, Apoptosis

## Abstract

**Background:**

Neural stem cells (NSCs) hold great potential for the treatment of neurodegenerative diseases. However, programmed cell death (PCD) provoked by the harsh conditions evident in the diseased brain greatly undermines the potential of NSCs. Currently, the mechanisms of PCD that effect NSCs remain largely unknown.

**Results:**

We have previously reported that hippocampal neural stem (HCN) cells derived from the adult rat brain undergo autopahgic cell death (ACD) following insulin withdrawal without hallmarks of apoptosis despite their normal apoptotic capabilities. In this study, we demonstrate that glycogen synthase kinase 3β (GSK-3β) induces ACD in insulin-deprived HCN cells. Both pharmacological and genetic inactivation of GSK-3β significantly decreased ACD, while activation of GSK-3β increased autophagic flux and caused more cell death without inducing apoptosis following insulin withdrawal. In contrast, knockdown of GSK-3α barely affected ACD, lending further support to the critical role of GSK-3β.

**Conclusion:**

Collectively, these data demonstrate that GSK-3β is a key regulator of ACD in HCN cells following insulin withdrawal. The absence of apoptotic indices in GSK-3β-induced cell death in insulin-deprived HCN cells corroborates the notion that HCN cell death following insulin withdrawal represents the genuine model of ACD in apoptosis-intact mammalian cells and identifies GSK-3β as a key negative effector of NSC survival downstream of insulin signaling.

## Background

Multipotent neural stem cells (NSCs) have the capacity to self-renew and generate differentiated progeny of neural cell types, such as neurons, astrocytes, and oligodendrocytes [1]. Increasing evidence suggests that adult NSCs progressively generate new neurons, even in adulthood, and that this adult neurogenesis contributes intrinsically to brain development, tissue homeostasis, and cognitive function [2]. However, the capability for adult neurogenesis debilitates with aging or neurodegeneration, and the size of the adult NSC pool decreases. This decrease raises the possibility that deregulated programmed cell death (PCD), as well as alterations in the rate of proliferation and differentiation of NSCs, may contribute to deteriorated neurogenesis in the diseased brain [3]. Indeed, PCD is important in regulating the size of the NSC population at various stages of neural development. However, the molecular mechanisms of PCD that underlie the debilitated capability of the adult NSCs during neurodegeneration remain poorly understood.

Different types of PCD are characterized by morphological and biochemical criteria and classified into three major types: apoptosis; autophagic cell death (ACD); and necrosis. Compared with the relatively well-studied physiological roles and biochemical mechanisms of apoptosis, the roles and relevant molecular mechanisms of ACD are largely unknown, especially in mammals. Our recent study revealed that hippocampal neural stem (HCN) cells derived from adult rats undergo ACD following insulin withdrawal [4-6]. HCN cells have intact apoptotic machinery; nevertheless, they undergo ACD with an increased autophagic flux upon insulin withdrawal. Of note, apoptotic hallmarks are absent in insulin-deprived HCN cells. Because the degree of cell death is proportional to the level of autophagy without apoptosis activation and knockdown of the key autophagy gene *Atg7* reduces cell death, insulin-deprived HCN cells meet the strict criteria suggested as definitive of ACD, and are considered as the most genuine model of ACD in mammalian systems [7,8].

Autophagy is an evolutionarily conserved catabolic process for degradation of cytosolic proteins and organelles by forming autophagosome for cargo loading and subsequent fusion with lysosomes [9]. Autophagy can be induced by a variety of stress stimuli, such as nutrient and growth factor deprivation, protein aggregation, mitochondrial damage, or pathogen infection [10]. A large body of literature has demonstrated the cytoprotective role of autophagy in sustaining cellular stress. Autophagy relieves cellular stresses by removing sources of stresses, such as toxic aggregated proteins, dysfunctional subcellular organelles, or infectious agents. Additionally, autophagy can contribute to fulfilling acute metabolic needs under starvation conditions by degrading and recycling the cargos. In opposition to these pro-survival roles, recent evidence including our own research, suggests that autophagy may also serve as an alternative, non-apoptotic mode of cell death referred to as ACD [11].

GSK-3 is a serine/threonine kinase that regulates a variety of cellular functions including glycogen synthesis, metabolism, proliferation, differentiation, apoptosis, insulin signaling, and decision of cell fates during embryonic development [12-15]. GSK3 exists in two isoforms, GSK-3α (51 kDa) and GSK-3β (47 kDa), each encoded by separate genes with an overall homology of 85% [16]. The two isoforms have highly conserved kinase domains, but differ at the N- and C-terminals. Additionally, the two isoforms of GSK-3 are not functionally identical, as demonstrated by embryonic lethality only in GSK-3β knockout mice [17,18]. Moreover, GSK-3β is found ubiquitously throughout the animal kingdom with particularly high levels in the central nervous system, whereas GSK-3α is expressed only in vertebrates [19]. Recent studies have suggested that GSK-3β plays critical roles in neural development, cell death, and the maintenance of pluripotency during neurodevelopment [20-22]. An additional well-explored aspect of GSK-3β is its role in neuronal death and neurodegeneration. GSK-3β activation leads to neuronal apoptosis, and the formation of amyloid plaques, the phosphorylation of tau proteins, and the formation of neurofibrillary tangles in models of Alzheimer’s disease [23,24].

GSK-3β is a downstream negative regulator of the insulin response and is inhibited by insulin signaling [25,26]. Given the role of GSK-3β in neuronal apoptosis and neurodegeneration [27-29], GSK-3β may be a critical regulator of cellular responses to stress, such as insulin withdrawal. These findings prompted us to propose the involvement of GSK-3β in regulation of ACD in HCN cells following insulin withdrawal. In this report, we found that insulin withdrawal triggered the activation of GSK-3β, suggesting that GSK-3β may play an important role in HCN cell death. Inhibition of GSK-3β using pharmacological inhibitor and gene silencing significantly decreased ACD. On the other hand, over-activation of GSK-3β through expression of wildtype (WT) or constitutively active (CA) forms of GSK-3β led to augmentation of ACD without inducing apoptosis. These results support the assertion that insulin withdrawal-induced death of HCN cells represents the genuine model of ACD in mammalian cells, and identify GSK-3β as a critical regulator of ACD in HCN cells.

## Results

### GSK-3β is activated in HCN cells following insulin withdrawal

**Figure 1:**
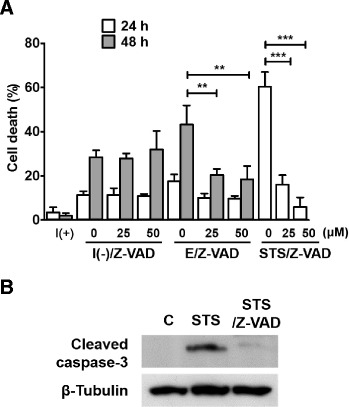
Caspase inhibition failed to protect HCN cells from insulin withdrawal-induced cell death. **(A)** Z-VAD.fmk failed to prevent insulin withdrawal-induced HCN cell death. On the other hand, apoptotic cell death induced by etoposide (E, 20 μM) or staurosporine (STS, 0.5 μM) in I(+) was effectively blocked by Z-VAD.fmk. Insulin presence and withdrawal were denoted as I(+) and I(−), respectively. Cell death was measured by PI/Hoechst staining and quantitative data are presented as the mean ± SD (n = 3). Statistical significance was determined with an ANOVA test by comparing within each treatment group with and without Z-VAD.fmk in the same day ***p < 0.001. **(B)** Inhibition of caspase 3 activation by Z-VAD.fmk. E, etoposide; STS, staurosporine; C, control.

In our previous reports, we demonstrated that HCN cells undergo a genuine ACD without signs of apoptosis upon insulin withdrawal [4,6]. Of note, HCN cells are subject to apoptosis in response to prototypical apoptosis inducers, such as staurosporine (STS). These findings indicate that insulin-deprived HCN cells adopt ACD as the primary mode of cell death despite their intact apoptotic capability. To confirm the non-apoptotic nature of HCN cell death induced by insulin withdrawal, insulin-deprived HCN cells were treated with a pan-caspase inhibitor Z-VAD.fmk. The insulin-withdrawn and insulin-containing conditions are denoted as I(−) and I(+), respectively, throughout this report. Caspase activation was not observed in HCN cells cultured in either condition. Consistent with this observation, Z-VAD.fmk failed to protect HCN cells from insulin withdrawal (Figure 1A). In sharp contrast to this ineffectiveness against insulin withdrawal, Z-VAD.fmk efficiently blocked apoptotic cell death induced by etoposide and STS in a dose-dependent manner (Figure 1A). Inhibition of caspase-3 activation by Z-VAD.fmk was confirmed by Western blotting analysis in apoptotic cells treated with STS (Figure 1B). The normal induction of apoptosis by prototype apoptotic inducers verified the intact apoptotic capability of HCN cells.

GSK-3β expression levels are particularly high in the brain [19], and broadly implicated in neuronal cell death and neurodegeneration [27-29]. Additionally, GSK-3β activation promotes apoptosis in various stress conditions, and over-expression of GSK-3β leads to neuronal apoptosis [29]. On the contrary, GSK-3β is inhibited by prosurival growth factors including insulin. Therefore, the pro-survival and proliferative effects of insulin signaling seem, at least in part, to be mediated through suppression of GSK-3β. Based on these findings, we hypothesized that GSK-3β may positively regulate the induction of autophagy and subsequent cell death in HCN cells following insulin withdrawal. Thus, an increase in GSK-3β activity should result in higher levels of autophagic flux and ACD. On the other hand, the opposite should occur when GSK-3β is decreased by pharmacological or genetic means. Herein, we addressed our hypotheisis by modulating GSK-3β activity in insulin-deprived HCN cells.

**Figure 2:**
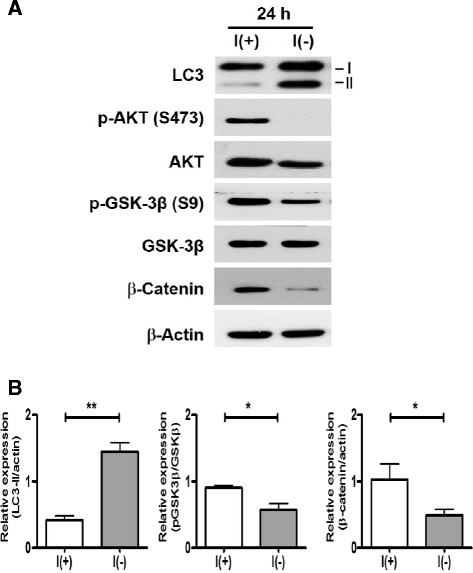
Insulin withdrawal activated GSK-3β. **(A)** Insulin withdrawal activated GSK-3β, as shown by the decrease in the inhibitory phosphorylation level of serine 9 and the reduction in the β-catenin level. Accompanied by inactivation of AKT, autophagy induction was manifested by an increase in the LC3-II level. Representative results from at least three independent experiments are shown. **(B)** Quantitative analyses of the LC3-II and β-catenin levels after normalization to β-actin, and phos-GSK-3β level after normalization to total-GSK-3β. Quantitative data are presented as the mean ± SD (n = 3). *p < 0.05, **p < 0.01. Statistical significance was determined with unpaired Student’s t- test.

First, to examine whether GSK-3β was activated following insulin withdrawal, we monitored the level of inhibitory phosphorylation at the GSK-3β serine 9 residue by Western blotting analysis. Stimulation of cells with insulin causes inactivation of GSK-3 via a phosphatidylinositol 3 kinase (PI3K)-dependent mechanism. PI3K-induced activation of protein kinase B (PKB; also known as AKT) phosphorylates serine 9 on GSK-3β and serine 21 on GSK-3α [26,30]. Phosphorylation of these residues inhibits GSK-3 activity. As expected, AKT was inactivated and phosphorylation levels of GSK-3β (Ser9) decreased in insulin-deprived HCN cells, indicating activation of GSK-3β (Figure 2A, B). As an additional indicator of GSK-3β activity, we also monitored the protein level of β-catenin, which is phosphorylated by GSK-3β and subsequently degraded through the ubiquitin-proteasome system [31]. As such, the β-catenin level was substantially decreased in insulin-deprived HCN cells (Figure 2A, B). Consistent with our previous reports, insulin withdrawal caused a significant increase in the level of the type II of microtubule-associated protein light chain 3 (LC3-II), the most reliable biochemical marker of autophagy (Figure 2A, B) [32]. In combination with our earlier work demonstrating the prevention of cell death by *Atg7* knockdown in HCN cells, these data revealed that ACD was the primary cell death mechanism in insulin-deprived HCN cells, which manifested increased levels of autophagy and GSK-3β activation.

### Pharmacological and genetic inhibition of GSK-3β decreases ACD in insulin-deprived HCN cells

**Figure 3:**
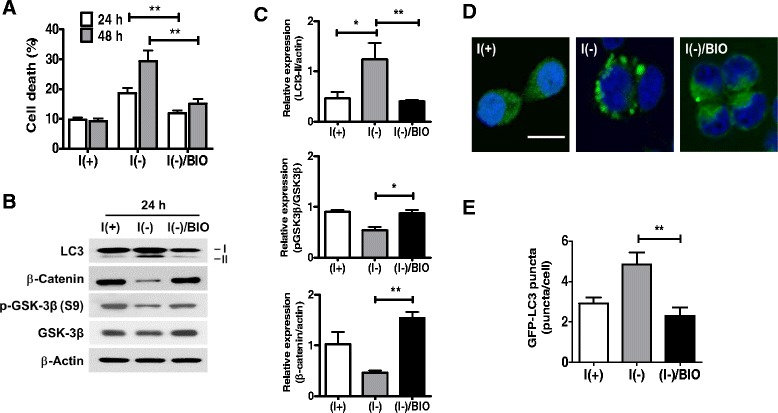
Pharmacological inhibition of GSK-3β protected HCN cells from insulin withdrawal-induced ACD. **(A)** The GSK-3 inhibitor BIO (0.25 μM) markedly decreased the cell death induced by insulin withdrawal. **(B)** BIO strongly inhibited GSK-3β activity, as shown by a restoration of the β-catenin level, and attenuated autophagic flux, as demonstrated by Western blotting analyses of the LC3-II level 24 h after insulin withdrawal. **(C)** Quantitative analyses of the LC3-II and β-catenin levels after normalization to β-actin, and phos-GSK-3β level after normalization to total-GSK-3β. Quantitative data are presented as the mean ± SD (n = 3). *p < 0.05, **p < 0.01. **(D)** The autophagic flux rate was estimated by GFP-LC3 punta assay in HCN cells by counting more than 50 cells from three independent experiments. The representative results are shown. Scale bar is 10 μm. **(E)** Quantitative data are presented as the mean ± SD (n = 3). **p < 0.01. Statistical significance was determined with an ANOVA test.

Given the role of GSK-3β in neuronal apoptosis, autophagy, and insulin signaling, we postulated that GSK-3β may regulate autophagy induction and cell death in HCN cells following insulin withdrawal. To test this, we used 6-bromoindirubin-3'-oxime (BIO), a specific GSK-3 inhibitor [33] which effectively prevented insulin withdrawal-induced cell death at a concentration of 0.25 μM as assessed by PI/Hoechst staining (Figure 3A). BIO strongly suppressed autophagy induction, as the increased level of LC3-II was lowered to the level comparable with the insulin-containing condition (Figure 3B, C) and the high number of the GFP-LC3 puncta observed in I(−) condition was significantly decreased (Figure 3D, E). Inhibition of GSK-3β activity was confirmed by the significant accumulation of β-catenin (Figure 3B, C). BIO itself had no effect on cell viability under the insulin-containing condition (data not shown).

**Figure 4:**
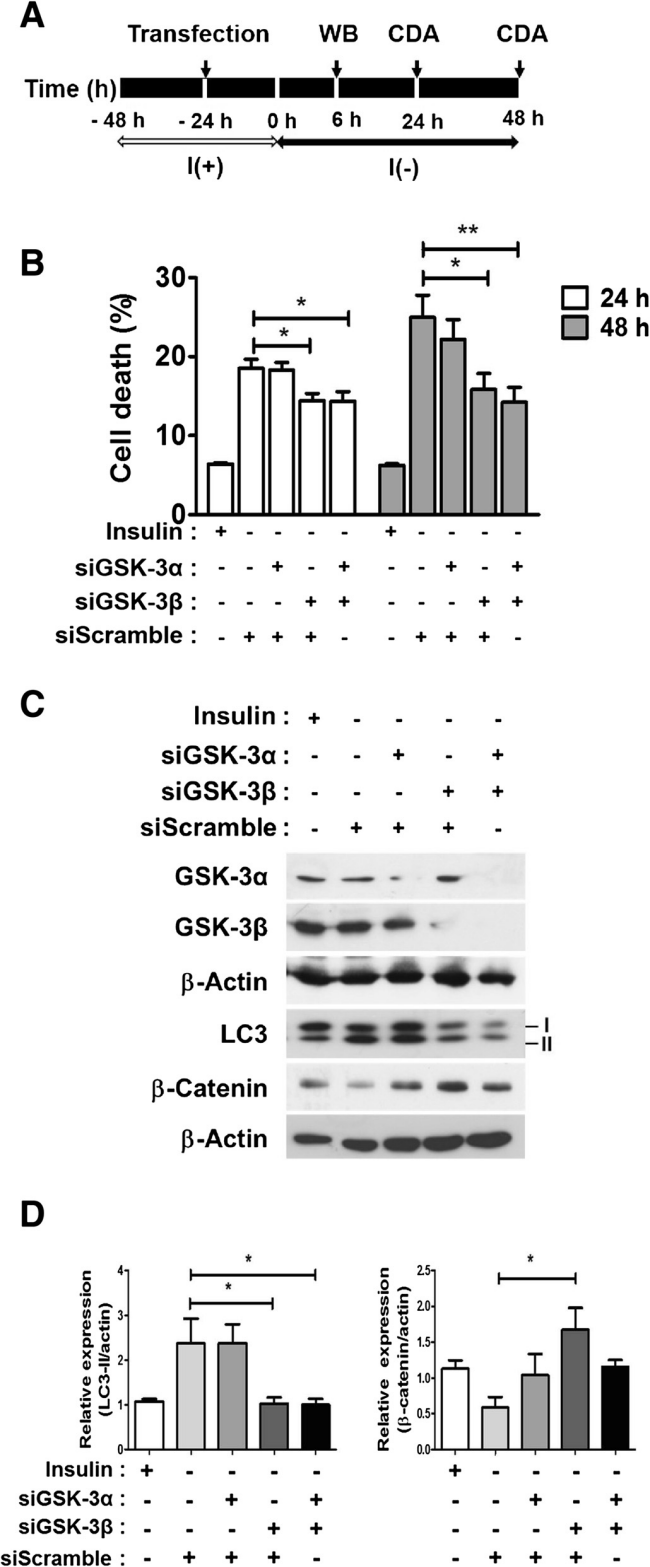
Knockdown of GSK-3β protected HCN cells from insulin withdrawal-induced ACD. **(A)** An experimental scheme for the knockdown of GSK-3 isoforms. CDA, cell death assay. **(B-C)** GSK-3β-targeting siRNA (30 nM) substantially decreased cell death (B), and attenuated autophagy after insulin withdrawal (C). Knockdown of GSK-3α barely affected ACD. Scrambled siRNA (30 nM) was added to GSK-3α or β-specific siRNA to keep the same total siRNA concentrations at 60 nM. Quantitative data are presented as the mean ± SD (n = 3). *p < 0.05, **p < 0.01. **(D)** Quantitative analyses of the LC3-II and β-catenin levels after normalization to β-actin. Quantitative data are presented as the mean ± SD (n = 3). *p < 0.05. Statistical significance was determined with an ANOVA test.

The pharmacologic inhibition of GSK-3 should affect both GSK-3α and -3β equally. To distinguish the relative contribution of the two isoforms to HCN cell death and demonstrate the essential role of GSK-3β in the regulation of ACD, we knocked down each isoform using specific small interfering RNAs (siRNAs; Figure 4A). In accordance with the pharmacological inhibition of GSK-3, GSK-3β siRNA attenuated insulin withdrawal-induced cell death (Figure 4B) and autophagy induction, as shown by a decrease in LC3-II level (Figure 4C, D). Of note, the GSK-3α-targeting siRNA yielded a marginal level of protection (Figure 4B), and double knockdown of both isoforms of GSK-3 led to a similar degree of the protective effect as observed for the GSK-3β single knockdown (Figure 4). These data supported our hypothesis regarding the critical role of GSK-3β in the regulation of ACD in HCN cells following insulin withdrawal.

### Over-expression of GSK-3β up-regulates autophagic flux and ACD, but not apoptosis, following insulin withdrawal in HCN cells

**Figure 5:**
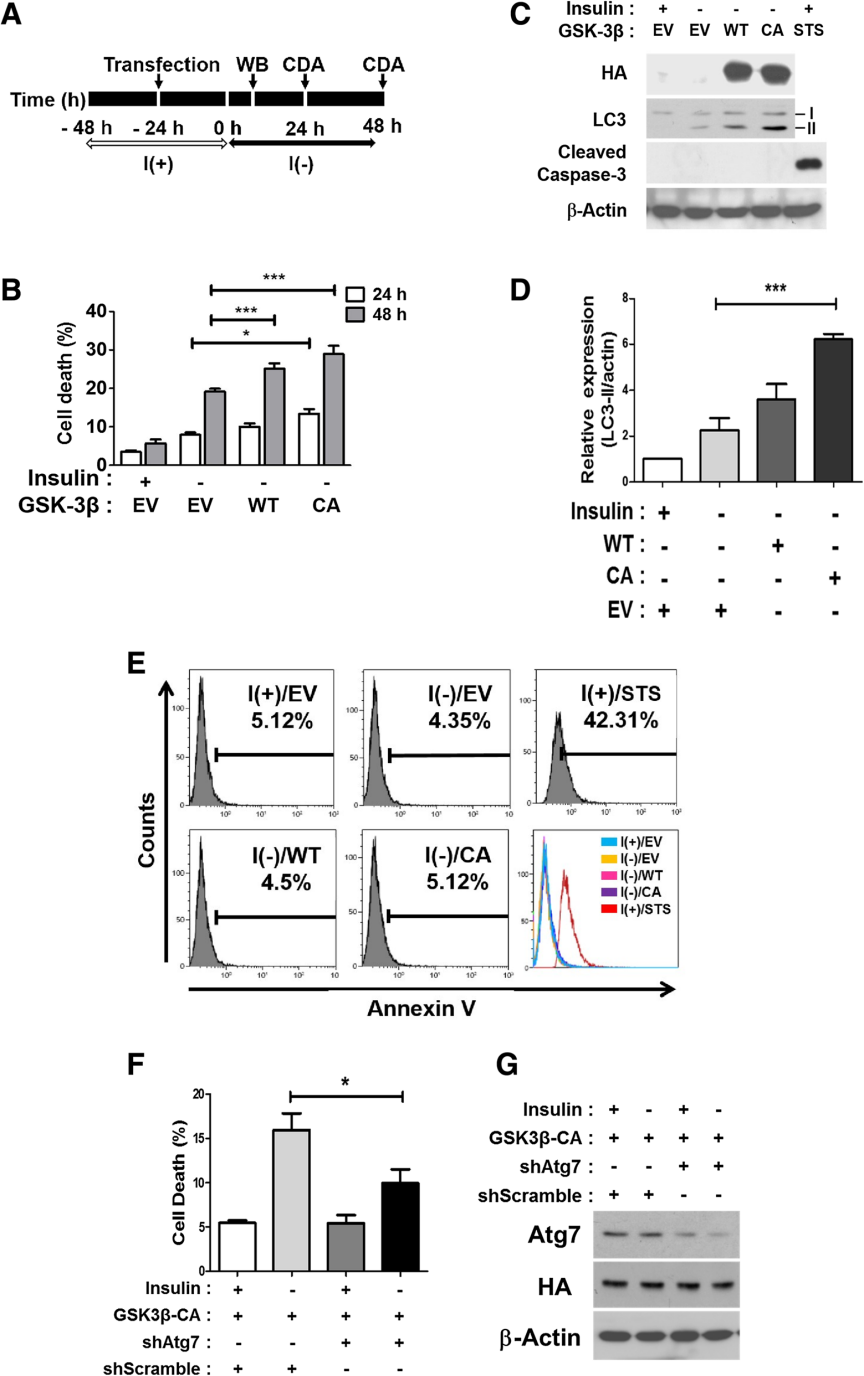
Over-expression of GSK-3β potentiated ACD without inducing apoptosis in insulin-deprived HCN cells. **(A)** An experimental scheme for the over-expression of GSK-3β constructs. CDA, cell death assay. **(B)** The GSK-3β WT and CA forms potentiated cell death, with more profound effects observed for the CA form in insulin-deprived HCN cells. EV, empty vector. **(C)** The potentiation of autophagy was well correlated with the elevated activities of GSK-3β. Alteration of the autophagy level was verified by Western blotting analyses of LC3-II 24 h after insulin withdrawal. Caspase-3 was not activated in spite of the increase in cell death in GSK-3β WT or CA-expressing cells. STS was treated for 6 h in I(+) as a positive control for caspase-3 activation. The over-expression of ectopic GSK-3β forms was confirmed by Western blotting analyses with an anti-HA antibody. **(D)** Quantitative analyses of the LC3-II level after normalization to β-actin. Quantitative data are presented as the mean ± SD (n = 3). ***p < 0.001. **(E)** The absence of apoptosis induction following GSK-3β over-activation was confirmed by Annexin-V staining and fluorescence-activated cell sorting analysis. Regardless of GSK-3β over-expression, the low percentages of Annexin-V-positive cells remained unaltered 24 h after insulin withdrawal. STS treatment for 6 h in I(+) was used to induce apoptosis as a positive control for Annexin-V staining and subsequent flow cytometry analysis. **(F)** Knockdown of Atg7 attenuated cell death. HCN cells were infected with pLKO.1- Atg7 shRNA and control scramble shRNA lentivirus. HCN cells with reduced level of Atg7 were transfected with GSK-3β CA form and cell death was measured 24 h after insulin withdrawal. Quantitative data are presented as the mean ± SD (n = 3). *p < 0.05. **(G)** Atg7 level and expression of HA-tagged GSK-3β CA were analyzed by Western blot analysis. STS, staurosporine.

To further establish the role of GSK-3β in the regulation of ACD in insulin-deprived HCN cells, we transfected HCN cells with the GSK-3β constructs encoding the WT and CA mutant forms using Lipofectamine method (Figure 5A). The CA form of GSK-3β was generated by mutation of the serine 9 residue to an alanine residue. This serine 9 residue is a key regulatory site for the inactivation of GSK-3β, and mutation of this residue to alanine prevents the inhibitory phosphorylation of the serine 9 residue, thereby avoiding enzyme inactivation [34,35]. Ectopic expression of the constructs was confirmed by Western blotting analysis with an antibody against the hemagglutinin (HA) epitope tag (Figure 5C). Over-expression of the GSK-3β WT form significantly increased cell death in insulin-deprived HCN cells (Figure 5B). In particular, the over-expression of GSK-3β CA mutant increased cell death to a greater extent than the WT form. Both the GSK-3β WT and CA forms elevated the level of LC3-II autophagy marker with more profound effects caused by the GSK-3β CA mutant form (Figure 5C, D). However, in spite of this marked increase in cell death, caspase-3 activation was not detected (Figure 5C). Furthermore, when apoptosis was monitored by Annexin V dye, which binds to the phosphatidylserine exposed on the outer leaflet of apoptotic cells, the low percentage of Annexin V-positive cells remained unaltered in the cells over-expressing the GSK-3β CA form, as well as the WT form, under insulin withdrawal condition for 24 h (Figure 5E). A high percentage of cells were positively stained by Annexin V after STS treatment for 6 h, verifying the accuracy of the assay. To confirm the autophagic nature of the GSK-3β-induced cell death, Atg7 was knocked down by infection of Atg7 shRNA-expressing lentivirus. Knockdown of Atg7 significantly attenuated cell death induced by over-expresion of GSK-3β CA (Figure 5F, G). Therefore, a lack of apoptosis indices despite a robust increase in LC3-II levels and cell death rates by GSK-3β provided further support for our hypothesis that GSK-3β was a critical upstream regulator of ACD in insulin-deprived HCN cells.

**Figure 6:**
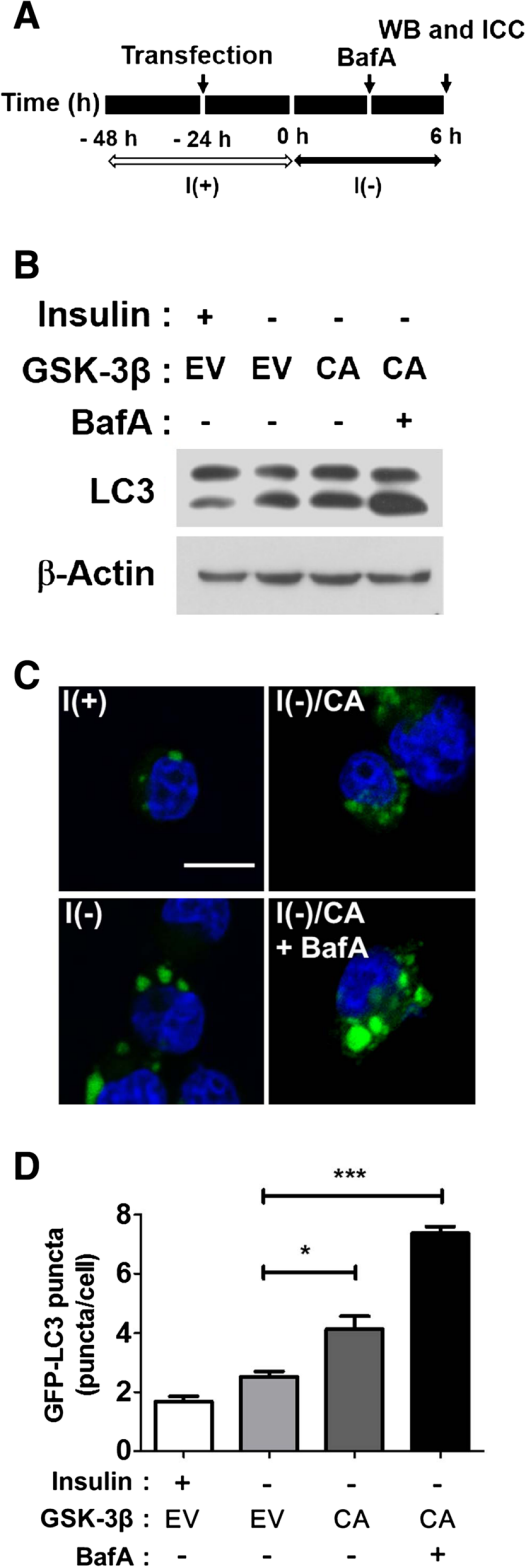
GSK-3β activation accelerated autophagic flux in insulin-deprived HCN cells. **(A)** An experimental scheme for the assessment of autophagic flux. **(B)** The autophagic flux rate was estimated by Western blotting analyses in GSK-3β CA-expressing HCN cells 6 h after insulin withdrawal. Bafilomycin A1 (BafA, 30 nM) was added 2 h before cell harvest. **(C)** The autophagic flux rate was estimated by GFP-LC3 punta assay in GSK-3β CA-expressing HCN cells 6 h after insulin withdrawal. BafA (30 nM) was added 2 h before imaging. The results shown are representative of three independent experiments. **(D)** Quantitation of GFP-LC3 puncta. More than 120 cells per condition were counted from three independent experiments. Quantitative data are presented as the mean ± SD (n = 3). Scale bar is 10 μm. *p < 0.05, ***p < 0.001. Statistical significance was determined with an ANOVA test. EV, empty vector, BafA, Bafilomycin A1.

In contrast to the absence of apoptosis, the autophagic flux rate was significantly boosted by high levels of GSK-3β activity. The furtherance of autophagic flux by the GSK-3β CA form was examined by analyses of the LC3-II protein levels and the GFP-LC3 puncta numbers in combination with the autophagic flux blocker, Bafilomycin A1 (BafA). An impairment of autophagy flux, as well as the induction of autophagy, which are opposite incidences in terms of autophagosome production and clearance, can lead to seemingly similar accumulations of LC3-II protein or LC3 puncta. Therefore, an increase in LC3-II levels or LC3 puncta numbers does not necessarily correlate with increased autophagic flux, and caution is required for the interpretation of results. A comparison between the steady-state levels of autophagosomes (before treatment with autophagic flux blockers, such as autophagosome-lysosome fusion inhibitors or intralysosomal protease inhibitors) and the accumulated levels of autophagosomes (after inhibitor treatment) has been recommended as a result. A high rate of autophagic flux would result in significantly different levels of autophagy markers between the steady state and accumulated autophagosome conditions, while marginal differences may denote an impaired autophagic flux [36]. An increase in the rate of autophagosome formation and clearance in insulin-deprived HCN cells was previously shown, demonstrating an increase of autophagic flux [4,6]. To measure the alteration in autophagic flux following over-expression of the GSK-3β CA form in insulin-deprived HCN cells, we used BafA which blocks the fusion between the autophagosome and lysosomes, preventing autophagic flux [37]. Ectopic expression of the GSK-3β CA form in I(−) led to greater increases in LC3-II level than insulin withdrawal alone (Figures 5C and 6B), and BafA treatment further boosted the increase in the LC3-II level induced by the GSK-3β CA form (Figure 6B). These results indicated that the GSK-3β CA mutant form accelerated autophagic flux more in insulin-deprived HCN cells, which already exhibited high levels of autophagic flux. An additional measure for assessing autophagic flux is the number of GFP-LC3 puncta. Autophagy activation induces a change in the GFP-tagged LC3 pattern from the diffused signal in the cytosol to a puncta/dot pattern following enrichment of LC3 in autophagosome [38]. Consistent with the notion of elevated autophagic activity, an increase in the number of GFP-LC3 puncta in the GSK-3β CA form-expressing HCN cells was facilitated to a greater extent by BafA treatment under the insulin-deficient condition (Figure 6C-D). Collectively, these data provided important evidence suggesting that GSK-3β may be the key regulator of ACD following insulin withdrawal in HCN cells.

## Discussion

Due to their potential for providing novel therapeutic avenues for the treatment of neurodegenerative diseases, multipotent NSCs have attracted considerable attention from both the scientific community and the public. However, the hostile environment in the aging or diseased brain greatly limits the proliferation and neurogenesis of NSCs and presents barriers to effective therapeutic design with engrafted or endogenous NSCs. Our present lack of knowledge regarding the molecular mechanisms governing the survival and death of NSCs during degeneration has hindered the utilization of NSCs for the treatment of brain diseases.

In this report, we demonstrated that GSK-3β was a critical upstream regulator of ACD in insulin-deprived HCN cells. We used two readouts to monitor GSK-3β activity; the inhibitory phosphorylation status of the serine 9 residue, and the β-catenin level. Activation of GSK-3β following insulin withdrawal augmented ACD, while pharmacological inhibition and gene silencing of GSK-3β attenuated ACD. The mode of cell death caused by GSK-3β activation was of special interest to us. Over-expression of GSK-3β is known to induce apoptosis in neurons and various other cell types. However, over-expression of the GSK-3β WT or CA mutant forms in insulin-deprived HCN cells gave rise to greater increases in autophagic flux and cell death as compared with insulin withdrawal alone, but without inducing apoptosis. The GSK-3β CA form accelerated the autophagic flux rate greatly, and caused more cell death than the WT form. These data demonstrated that the status of GSK-3β activation was well correlated with the level of ACD, but that there was no induction of apoptosis regardless of the activities of GSK-3β in HCN cells following insulin withdrawal. Given the apoptotic role of GS-3β in other cell types, it is an intriguing question to ask how two different modes of cell death can be distinguished and what signals are specific to ACD in relation to GSK-3β in HCN cells. One potential mechanism may be related with calpain activation. Recently, Jin et al. reported the cleavage of GSK-3β by calpain can potentiate the neurotoxic activity of GSK-3β [39]. It is conceivable that further modification of GSK-3β may be required to turn its autophagic activity into apoptosis. Collectively, the results of our study established GSK-3β as a major modulator of ACD in insulin-deprived HCN cells.

The prime role of autophagy at the basal state is to protect cells from stress conditions by degrading damaged organelles and proteins, or by supplying metabolic provisions and biochemical intermediates. However, emerging evidence suggests that autophagy may play a causative role in cell death when it is induced excessively. The growing interest in the molecular mechanisms of ACD was highlighted by a recent debate focused on the definition and roles of ACD [7,40,41]. Further studies into the molecular mechanisms of induction and execution of ACD and its interaction with apoptosis are urgently warranted in order to resolve the current controversies and advance our understanding of this intriguing mode of cell death.

Though GSK-3β activation has been widely implicated in cell death, GSK-3β is a bifunctional enzyme that plays conflicting roles by promoting cell survival under certain conditions [12]. In HCN cells, activation of GSK-3β following insulin withdrawal seems to be detrimental rather than protective, as the inhibition and genetic suppression of endogenous GSK-3β abated the autophagy level and subsequent cell death. On the other hand, over-expression of the GSK-3β WT form substantially elevated ACD, and the CA form exhibited an even more potent effect.

NSC transplantation has been a popularly conceived therapeutic approach to treat various devastating neurodegenerative diseases. However, the use of exogenous stem cells has been associated with barriers based on ethical and technical issues. Therefore, utilization of endogenous NSCs can be an ideal alternative. In order to optimize the therapeutic potential of endogenous NSCs, it will be essential to understand how NSCs respond to adverse cellular stresses and identify the signaling molecules involved in the regulation of NSC function, especially the signaling cascades governing the death of NSCs [3]. The molecular mechanisms of PCD that affect the NSC population remain obscure. In HCN cells, the apoptotic machinery is intact. Therefore, the drivers of the mode of cell death provoked by insulin withdrawal and GSK-3β activation that results in a preference of ACD over apoptosis is particularly intriguing. Much remains to be learned to address this stimulating question, but the efforts required to pursue such answers will surely provide novel insights into the role of autophagy and PCD in NSC biology.

## Methods

### Cell culture and chemicals

HCN cells were cultured as previously described [6]. Stock solutions of Z-VAD.fmk (BD Pharmingen, Franklin Lake, NJ, USA), Bafilomycin A1 (Sigma-Aldrich, St. Louis, MO, USA) and BIO (Sigma-Aldrich) were prepared in dimethyl sulfoxide at appropriate concentrations.

### Cell death assay

HCN cells were seeded in 96-well plates at a cell density of 1.0 × 10^5^ cells/mL. Hoechst 33342 and propidium iodide (PI; Invitrogen, Waltham, MA, USA) stock solutions were diluted with phosphate-buffered saline (PBS). After adding the diluted Hoechst and PI solutions to the wells (1% volume of media in the well, final 1/1000 dilution) in the dark, plates were incubated for 20 min at 37°C. Blue and red signal-positive cells were counted under fluorescence microscopy. The percentage of cell death was calculated as follows:

$$ \mathrm{Cell}\ \mathrm{death}\ \left(\%\right) = \left(\mathrm{PI}\ \left[\mathrm{red}\right]\ \mathrm{positive}\ \mathrm{cell}\ \mathrm{number}/\mathrm{total}\ \mathrm{cell}\ \mathrm{number}\ \left[\mathrm{blue}\right]\right) \times 100 $$

### Annexin V staining

Annexin V-FITC (BD Biosciences, San Jose, CA, USA) was used for the determination of apoptosis. HCN cells were harvested 24 h after insulin withdrawal by trypsin-EDTA and centrifugation at 500 g for 5 min at 4°C. After cells were labeled with the Annexin V–FITC (1: 500 dilution) for 15 min at room temperature, samples were analyzed by flow cytometry using a Gallios Flow Cytometer (Beckman Coulter, Brea, CA, USA). Data were analyzed using Kaluza software.

### Transfection

pEGFP-LC3 and ON-TARGET PLUS siRNAs specific for rat GSK-3α and GSK-3β sequences were purchased from Addgene (Cambridge, MA, USA) and Dharmacon (Lafayette, CO, USA), respectively. HCN cells were transfected with GSK-3β constructs and siRNA using Lipofectamine 2000 (Invitrogen) according to the manufacturer’s instructions. Transfection was performed in culture medium without insulin and penicillin/streptomycin. After 2 h, the transfection media was replaced with culture medium.

### GFP-LC3 puncta assay

The GFP-LC3-transfected HCN cells were plated on glass coverslips in 24-well plates at a cell density of 2.0 × 10^5^ cells/mL. The HCN cells were fixed in 4% paraformaldehyde (PFA) solution for 10 min at room temperature. After removal of PFA and rinsing in PBS twice, the cells were mounted on slides with Mount solution (Dako, Glostrup, Denmark) and the images of the GFP-LC3-positive cells was obtained with a confocal microscope (Cal Zeiss LSM700). The automated algorithmic quantification of the number and size of GFP-LC3 puncta was performed using the custom ImageJ macro called GFP-LC3 macro [42]. The cut-off size of the puncta was set at 0.25 μm with the roundness values between 0 and 1.5.

### Western blot analysis

The HCN cells were harvested, and lysates were prepared using lysis buffer (250 mM sucrose, 50 mM NaCl 1%, TritonX-100, 1 mM dithiothreitol, 1 mM phenylmethylsulfonyl fluoride in 20 mM Tris–HCl, pH7.2) containing 1× protease cocktail inhibitors (Pierce, Rockford, IL, USA) and 1× phosphatase cocktail inhibitors (Pierce). Protein concentration was determined by a BCA protein assay kit (Pierce). Proteins were loaded into the gel and electrotransferred to a polyvinylidene fluoride membrane with a Trans-Blot SD Semi-Dry Electrophoretic Transfer Cell (Bio-Rad, Hercules, CA, USA). Then, membranes were blocked for 1 h at room temperature in a blocking solution of 5% nonfat dry milk in 1× Tris-buffered saline with 0.1% of Tween 20. Membranes were incubated overnight with primary antibodies. Primary antibodies were used as follows: total Akt, phosphorylated Akt (serine 473), β-actin, β-catenin, Bcl-2, total GSK-3β and α, phosphorylated GSK-3β (serine 9) from Cell Signaling Technology (Danvers, MA, USA); and LC3 (Sigma-Aldrich). After washing with blocking solution, the membranes were incubated for 1 h at room temperature in blocking solution containing peroxidase-conjugated secondary antibodies. After washing the membranes, protein expression was analyzed by using a supersignal chemiluminescence detection kit (Pierce).

### Lentiviral production and concentration

Lentivirus vector pLKO.1 scramble shRNA (plasmid 1864), enveloper vector pMD2.G (plasmid 12259), and packaging vector psPAX2 (plasmid 12260) were obtained from Addgene. pLKO.1 Sh-Atg7 vector (Sigma-Aldrich TRCN0000092164, TRCN0000369085) was purchased for Sigma. Lentiviruses were produced according to the manufacturer’s instruction and concentrated with polyethylene glycol 6000 (Sigma-Aldrich) after centrifugation for 30 min at 2,500 g and re-suspended in insulin-deficient medium. The HCN cells were infected for 12 h and the medium was replaced with fresh, virus-free medium. After 48 h incubation, the medium was replaced with medium containing 5 μg/ml puromycin for selection. Next day, the surviving cells were pooled for the experiment.

### Statistical analysis

All data values are presented as mean ± standard deviation (SD), and were obtained by averaging the data from at least 3 independent experiments. Statistical significance was determined by the unpaired Student’s t-test or one-way analysis of variance (ANOVA) followed by Tukey’s multiple comparison tests using GraphPad Prism (GraphPad Software, San Diego, CA, USA).

## Abbreviations

BafA: Bafilomycin A1
CA: Constitutively active
GSK: Glycogen synthase kinase
HA: Hemagglutinin
HCN cell: Hippocampal neural stem cell
LC3-II: The type II of microtubule-associated protein light chain 3
NSC: Neural stem cell
PBS: Phosphate-buffered saline
PCD: Programmed cell death
PFA: Paraformaldehyde
PI: Propidium iodide
PI3K: Phosphatidylinositol 3 kinase
PKB: Protein kinase B
siRNA: Small interfering RNA
STS: Staurosporine
WT: Wildtype
